# Exploring the usefulness of interviewers’ training before and after Multiple Mini Interviews (MMI) for undergraduate medical students’ selection: Was it really helpful?

**DOI:** 10.12669/pjms.326.11175

**Published:** 2016

**Authors:** Sobia Ali, Hasan Shoaib, Rehana Rehman

**Affiliations:** 1 Dr. Sobia Ali, MHPE. Department of Medical Education, Shalamar Medical and Dental College, Lahore, Pakistan; 2 Dr. Hasan Shoaib, DCPS-HPE. Department of Medical Education, University of Lahore, Lahore, Pakistan; 3 Dr. Rehana Rehman, PhD. Department of Biological and Biomedical Sciences, Agha Khan University, Karachi, Pakistan

**Keywords:** MMI assessors’, training, Faculty training evaluation

## Abstract

**Objective:**

To compare the change in interviewers’ perception of Multiple Mini Interviews (MMI) after MMI training and after actual MMI experience.

**Methods:**

Six sessions were conducted during two weeks (October 26, 2015- to November 6, 2015) to a total of 87 faculty members. The evaluation dealt with 13 items questionnaire for representation of assessors’ perception on 5 point rating scale. Assessors rated their perceptions to complete an anonymised questionnaire about rationale behind MMI, the process of MMI, and the use of scoring criteria (rubrics). In addition, assessors were also asked to rate their level of satisfaction on MMI process after training and after interviews. Wilcoxon Signed Ranks Test (two-tailed) was used to compare participant’s pre- and post-interview ratings.

**Results:**

With 81.6% response rate, the positive views of assessors about the MMI selection process and the use of scoring criteria (Rubric) to assess the candidate are not altered after experiencing a MMI selection day (p> 0.001). Assessors (87%) would prefer to be involved in the process of MMI in future.

**Conclusion:**

The outstanding consistency of assessors’ ratings before and after interview concluded that MMI training sessions were helpful in improving knowledge and skills about MMI process and candidates’ assessment criteria (rubrics).

## INTRODUCTION

Multiple Mini Interviews (MMI) have been widely used for selection of students in health professions program worldwide.^1^-^3^ This process caters the assessment of non-cognitive and/or higher cognitive attributes in prospective students^4^,^5^ and have also been proved to have robust psychometric properties.

Multiple studies have addressed the factors that are responsible for its high psychometric properties and these include: blueprinting, its structural and functional organization, quality of stations, marking scheme and assessors’ training.^6^-^9^ There is evidence of variance between individual interviewers’ ratings^6^,^10^ due to gender and interviewer personality type and that is why the skill based training is strong recommendation as it helps in reducing the errors related to interviewers’ subjectivity, and characteristics.

Repeated workshops are the most common strategy used for capacity building in any context.^11^ Pre-test followed by post-test type evaluations are one of the various methods to evaluate the effectiveness of workshops in various settings. This method is frequently criticized because of the presence of response-shift bias in self-assessment.^12^ Similarly, ratings taken immediately only after training are also not favoured as the true outcome cannot be claimed through this.^13^ Literature in faculty development also exhibits that measurement of the outcomes & the measurement of true impact is difficult and questions about trainings’ effectiveness have been raised continuously and perceptions without actual performance make the training questionable.^11^ As a solution to these issues and assuming that participants’ perception after actual experience would help in concluding the effectiveness of training sessions, we designed a study in which we asked the assessors to rate their perceptions about process, training and skills achieved by the training immediately after the training and then again after actual experience of MMI to see the difference in their rating that would help gauge the effectiveness of MMI assessors’ training.

We also found that data is available on effectiveness of workshops but specifically related to MMI training is not available^14^ especially in our context. Similarly the importance of interviewers’ training have been emphasized in about every article discussing the process of development of MMI in one’s own context but no data is available on assessing the effectiveness of these trainings from interviewers’ point of view. These facts lead to the rationale behind this study with the objective to compare the change in interviewers’ perception of MMI after MMI training and after actual MMI experience.

## METHOD

All interviewers were invited to attend a three hour training session, 15 days prior to MMI from October26, 2015 to November 6, 2015.

### Training Session Participants

Six sessions were conducted for 87 faculty members of Shalimar Medical & Dental College (SMDC). Both clinical and basic sciences faculty were invited for each session. The training was attribute specific i.e. each group got the training to assess a single attribute (identified in blueprinting process).

### Training Outline

The interactive training session focussed on understanding the rationale and the process of MMI and use of rubric (marking criteria for subjective assessment) and score sheet to rate the applicants.

The first 40 minutes were predominantly theory based about the rationale behind MMI, information about the detail of the process of MMI, the detail of identified attributes (that were going to be assessed through MMI), the use of rubric for scoring the applicant’s behaviour and the use of score sheet for marking purpose. After a short break, the next session dealt with the practical training of the process, in which the participants (interviewers) were asked to practice their skill by viewing and assessing two role plays addressing the same attribute. The role plays were performed by trained actors simulating the situation of interview. The participants were then asked to rate the applicant on the basis of rubrics provided to them for a specific attribute dedicated to assess at a single station. In this manner they got practice of using the score sheet along with the rubric.

### Data Collection

At the end of each training session, faculty were asked to give their perception about usefulness of training session on an anonymised mix questionnaire. The perceptions from the trainees were taken after training (pre-experience) and after actual MMI experience (post-experience) to assess the training effectiveness.

The close ended questions dealt with understanding of rationale and process of MMI through training and use of rubrics, scoring sheet and level of satisfaction with the process of MMI. There were 13 items in this section. A rating scale format was used to elicit each interviewer’s responses to the item, where 1= the least and 5= the most. Most liked, least liked aspects and suggestions for improvement were also asked in the form of open ended questionnaire.

### Statistical Analysis

Data was analysed by SPSS version 20. Analysis methods included computing descriptive statistics about gender, speciality, designation, specific attribute for which they got the training and Cronbach’s alpha for internal consistency of the questionnaire (pre and post-experience). Nonparametric Wilcoxon Signed Ranks Test (two-tailed) was used for comparing paired pre and post-MMI ratings of interviewers because of the skewed data.

## RESULTS

A total of 87 faculty members attended six workshops (each target single specific attribute). Seventy one (81.6%) were those who filled both post-training and post-MMI questionnaires. Out of them 39 (54.9%) were males, 32 (45.1%) were females. The responses were relatively higher from clinical faculty compared to basic sciences faculty (Table-I).

**Table-I:** Gender, specialty and response distribution by training session attended (n=71).

Participants’ Gender	n (%)
Male	39 (54.9%)
Female	32 (45.1%)
*Specialty*	
Basic sciences	30 (42.2%)
Clinical sciences	41 (57.8%)
*Participants response distribution according to the training session for specific attribute*	
Communication Skills	16 (22.5%)
Critical Thinking	13 (18.3%)
Empathy	12 (16.9%)
Ethical Decision Making	15(21.1%)
Motivation	2(2.8)
Team Work	13 (18.3%)

The 13 items were analysed for internal reliability using Cronbach’s alpha, which yielded a reliability coefficient of Cronbach’s alpha 0.882 and 0.941 before and after actual MMI experience respectively. Changes in interviewers’ pre- MMI and post- MMI ratings for the workshops evaluation shows overall consistency in their responses (after program ratings minus before program ratings. Table-II. Using Wilcoxon Signed Ranks Test (two-tailed), we didn’t found a statistically significant change for interviewers’ rating scores of their training sessions’ effectiveness except for item 11 asking about clarity of stations’ instructions, that is showing significant difference (p<0.05). However, the trend of high interviewers’ rating is prominent on all items on both pre and post MMI experience for all workshops.

**Table-II:** Comparison of post-training and post-MMI rating of faculty.

		Negative Ranks		Positive Ranks		p-value

		Mean Rank	Sum of Ranks	Mean Rank	Sum of Ranks	
1	The interviewer training helped me understand the rationale for implementing the MMI	14.33	258.00	14.80	148.00	0.190
2	The interviewer training helped me understand the MMI process	18.47	314.00	16.53	281.00	0.758
3	Watching or participating in role play of MMI stations as a part of the interviewer training helped me to better understand the MMI process	17.87	268.00	15.29	260.00	0.938
4	Participating in the group discussion with other interviewers during training of MMI helped prepare me for my role as an interviewer	15.16	242.50	13.63	163.50	0.343
5	The prompt questions/rubrics helped me assess the primary attribute being evaluated	16.89	236.50	15.26	259.50	0.815
6	I was able to effectively differentiate between applicants	15.27	168.00	17.86	393.00	0.036
7	Every applicant had an equal opportunity to demonstrate the attribute being assessed	27.00	405.00	22.59	723.00	0.073
8	Five minutes was enough time for me to assess the attribute I was evaluating.	20.88	271.50	20.31	548.50	0.051
9	Two minutes was enough time for me to complete the evaluation form between applicants	16.50	165.00	14.21	270.00	0.234
10	The rubrics/criteria and assessment form for applicants was clear and easy to use	16.17	194.00	16.70	334.00	0.175
11	Instructions given to candidates before the station were clear enough	15.39	138.50	20.78	602.50	0.000*
12	MMI is the fair process of assessing higher cognitive and/or higher cognitive attributes of candidates	18.88	226.50	19.06	476.50	0.046
13	I would prefer to be involved in the process of MMI in future	12.89	116.00	13.82	235.00	0.103

## DISCUSSION

The problem of raters’ leniency in medical school selection interview was a factor leading to development of MMI.^10^ However, even after the development of such objective assessment, the interviewer bias remains the focal point of discussion and evidence always emphasises on interviewers’ training.^10^,^15^

The results of our study shows that higher rating trend of interviewers was consistent on both occasions with no significant difference on both occasions explains that usual tendency of workshops’ participants to rate high immediately after the workshops doesn’t change even after actual experience of performance as interviewers. We use this design for evaluating our workshops utility because we believe that this comparison provides a more delicate and effective evidence of the changes associated with this type of training than traditional pre- and post-intervention comparison. Basic philosophy behind adopting this approach is based on published studies that claimed low validity of traditional pre/post self-assessment results.^16^-^18^

“Instructions given to candidates before the station were clear enough” was the only item in our questionnaire which improves its rating significantly (positive mean ranks minus negative mean ranks, p<0.05). The obvious reason behind this change is that during the training sessions, participants’ were asked to give recommendations about process improvements and it was their strong recommendation to make the written instructions clear and easy to understand. Based on participants’ recommendations, the station authors reviewed all the written instructions and modified them to make them easily comprehensible.

The important finding in our study is that interviewers were highly satisfied with the training and agreed that training helped them understand the process and prepare them for their role as interviewers in MMI. In addition, they got their selves acquainted with the use of rubrics and score sheet for candidate’s assessment day. This is in accordance with study conducted by Hofmeister et al.^19^ that claim the same about interviewers’ satisfaction with the training. They felt they had enough time to assess the candidates at each station and agreed that this was a fair assessment of selecting medical students.

This follow up self-rating process in our data collection made up of single short intrusive questionnaire. This type of data collection also helps to minimize the response shift bias that is present in pre/post self-assessment data collection used generally for workshop evaluation because of inherent pre-test over or underestimation.^20^ This is the first study that evaluates the interviewers’ training for conducting MMI and also took feedback to improve the items.

### Limitations of the study

Our data encompasses the single cohort of faculty from single institute which restricts the generalizability of our results. With more research in this area our data will definitely strengthen the evidence. Secondly, this study didn’t show the analysis of qualitative results. Ongoing research by the authors about qualitative analysis will further refine the results.

## CONCLUSION

The consistency of interviewers’ rating (pre and post- MMI), and the published evidence against pre and post training self-assessment helped in conclusion that our design of follow-up post-performance rating (after actual required performance) is a valuable measure of the impact of the interviewers’ training. This also concluded that MMI training sessions were helpful in improving assessors’ knowledge and skills about MMI process and candidates’ assessment criteria (rubrics) and also helpful in retention of concepts gained during the training.
